# Motor Impairments in Transient Ischemic Attack Increase the Odds of a Subsequent Stroke: A Meta-Analysis

**DOI:** 10.3389/fneur.2017.00243

**Published:** 2017-06-07

**Authors:** Neha Lodha, Jane Harrell, Stephan Eisenschenk, Evangelos A. Christou

**Affiliations:** ^1^Department of Health and Exercise Science, Colorado State University, Fort Collins, CO, United States; ^2^Department of Applied Physiology and Kinesiology, University of Florida, Gainesville, FL, United States; ^3^Department of Neurology, University of Florida, Gainesville, FL, United States

**Keywords:** transient ischemic attack, motor impairments, stroke, odds ratio, meta-analysis

## Abstract

**Background and purpose:**

Transient ischemic attack (TIA) increases the risk for a subsequent stroke. Typical symptoms include motor weakness, gait disturbance, and loss of coordination. The association between the presence of motor impairments during a TIA and the chances of a subsequent stroke has not been examined. In the current meta-analysis, we examine whether the odds of a stroke are greater in TIA individuals who experience motor impairments as compared with those who do not experience motor impairments.

**Methods:**

We conducted a systematic search of electronic databases as well as manual searches of the reference lists of retrieved articles. The meta-analysis included studies that reported an odds ratio relating motor impairments to a subsequent stroke, or the number of individuals with or without motor impairments who experienced a subsequent stroke. We examined these studies using rigorous meta-analysis techniques including random effects model, forest and funnel plots, *I*^2^, publication bias, and fail-safe analysis.

**Results:**

Twenty-four studies with 15,129 participants from North America, Australia, Asia, and Europe qualified for inclusion. An odds ratio of 2.11 (95% CI, 1.67–2.65, *p* = 0.000) suggested that the chances of a subsequent stroke are increased by twofolds in individuals who experience motor impairments during a TIA compared with those individuals who have no motor impairments.

**Conclusion:**

The presence of motor impairments during TIA is a significantly high-risk clinical characteristic for a subsequent stroke. The current evidence for motor impairments following TIA relies exclusively on the clinical reports of unilateral motor weakness. A comprehensive examination of motor impairments in TIA will enhance TIA prognosis and restoration of residual motor impairments.

## Introduction

Transient ischemic attack (TIA) is a brief neurological event caused by temporary ischemia without acute infarction (1). Clinical symptoms include motor and speech impairments (2). These focal neurological symptoms are assumed to be resolved within 24 hrs. leaving no permanent damage to the central nervous system (3). Despite this, the risk for stroke increases up to 20% following a TIA (4). Nevertheless, the association between the presence of motor impairments during a TIA and a subsequent stroke is not well understood.

Interestingly, clinicians intuitively believe that motor impairments during a TIA predispose individuals to a greater risk for stroke. However, this clinical proposition lacks empirical validation. To-date, no study has quantified the influence of motor impairments during a TIA on the odds of a subsequent stroke. Therefore, the purpose of the current meta-analysis is to examine whether the odds of a subsequent stroke are greater in individuals who experience motor impairments during a TIA compared with those who do not experience motor impairments.

Typical motor impairments associated with TIA include unilateral motor weakness, paralysis of limbs, gait disturbance, and loss of coordination (2). However, clinical reports on motor impairments during a TIA have focused primarily on decreased muscle strength or motor weakness. A primary reason for the clinical focus on motor weakness is that weakness is easily examined in clinics as reduced muscle force during manual motor testing (5). Further, the pathophysiological rationale for assessing motor weakness is that it reflects upper motor neuron dysfunction associated with TIA and is a common accompaniment of other motor impairments (6, 7). Therefore, in the current meta-analysis, we use motor weakness as the primary measure of motor impairment during a TIA.

Several prognostic scores have been used to predict the risk of stroke after TIA (8) These scores are based on clinical characteristics of the patient such as age, blood pressure, clinical symptoms of motor weakness or speech impairment, duration of symptom, and diabetes. More recently, diagnostic neuroimaging has increased the predictive power of the clinical scores (9). Undoubtedly, the cumulative scores based on clinical and imaging characteristics provide useful information for TIA management. However, the unique contribution of motor impairments to increased risk for stroke has not been determined.

We use rigorous meta-analytic techniques to determine the extent to which the presence of motor impairments influences the likelihood of a subsequent stroke. Data from 24 studies with 15,129 participants from North America, Australia, Asia, and Europe are extracted and submitted to a meta-analysis. The current meta-analysis aims to precisely quantify the influence of motor impairments during a TIA on the risk for a subsequent stroke and inform clinicians, policy makers, and public educators on the importance of identifying and recognizing motor impairments in TIA.

## Materials and Methods

### Study Inclusion and Exclusion Criteria

**Figure 1:**
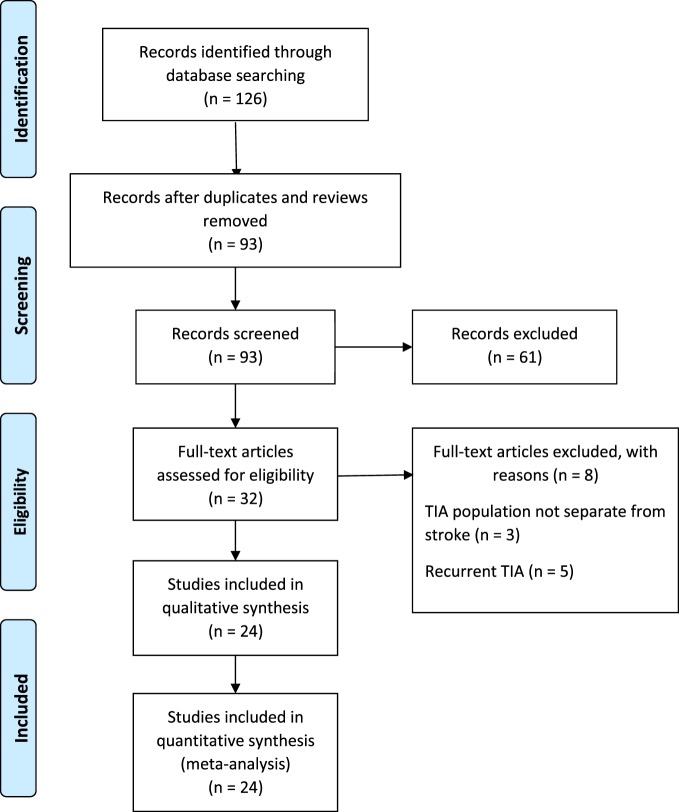
Study selection. Literature search and screening process.

The PRISMA guidelines directed the search and reporting of this meta-analysis. We conducted an exhaustive search for TIA studies using four computerized databases: (a) MEDLINE, (b) ISI’s Web of Knowledge, (c) Cochrane Database of Systematic Reviews, and (d) PsycINFO from July 1989 to December 2016. Fourteen key words and phrases dictated the search: TIA, stroke recurrence, motor deficit, weakness, hemiparesis, limb function, unilateral weakness, coordination, impairments, walking, gait disturbance, ataxia, dysarthria, and physical limitations. Additional search strategy involved manual searches to examine the reference lists of retrieved articles. Our initial search identified 126 records that discussed motor impairments in TIA and a subsequent stroke. We excluded the systematic reviews and studies that used the same population as another included study. Ninety-three unique records remained for additional screening. Figure 1 describes the literature search and screening process.

The inclusion/exclusion criteria were the following: (1) the study reported an odds ratio relating motor impairments to the chances of a subsequent stroke, or the number of individuals with and without motor impairments who experienced a subsequent stroke. Thirty-two of the original 93 studies met this criterion. (2) If studies evaluated multiple populations, such as minor stroke and TIA, then data for TIA population was reported separately. Three studies did not meet this criterion (10–12). (3) The recurrent ischemic event was required to be a stroke, not a recurrent TIA. Five papers failed this criterion (13–17). Twenty-four studies remained and were submitted to the meta-analysis (18–41). Two authors (Neha Lodha and Jane Harrell) independently coded and extracted data. A divergent evaluation was resolved in consultation with a third investigator (Evangelos A. Christou). All three investigators confirmed data extractions. All authors participated in the interpretation of the meta-analytic results. Table 1 describes the design and setting of the studies included in the meta-analysis.

**Table 1:** Design and setting of studies included in the meta-analysis.

Study name	Study period	Country	Clinical setting	Ascertainment method	Transient ischemic attack (TIA) diagnosed by	Follow-up
Al-Khaled and Eggers (18)	2007–2010	Germany	Multiple EDs	Prospective (consecutive referrals to stroke registry)	Neurologist, internist	Questionnaire
Ay et al. (19)	2000–2006	USA	Single ED	Retrospective (review of inpatient/outpatient reports)	Neurologist	Medical records
Bray et al. (20)	2004	Australia	Single ED	Retrospective (admissions to ED)	ED physician	Medical records and phone
Chandratheva et al. (21)	2002–2007	England	Multiple EDs and clinics	Prospective (multiple search methods)	Neurologist	In person
Chen et al. (22)	2006–2009	China	Multiple hospitals	Prospective (admissions to hospital)	Neurologist	In person
Coutts et al. (23)	Undetermined	Canada	Hospital stroke center	Prospective (referral to stroke team)	Stroke neurologist	In person
Dai et al. (24)	2009–2013	China	University hospital	Prospective (stroke registry)	Neurologist	In person or phone
Fujinami et al. (25)	2008–2009	Japan	Multiple stroke hospitals	Retrospective (review of medical records)	Attending physician	Medical records
Gon et al. (26)	2006–2013	Japan	Hospital stroke unit	Retrospective (review of medical records)	Not listed	Not listed
Hayashi et al. (27)	2007–2010	Japan	Single ED	Retrospective (review of medical records)	Neurologist	Medical records
Johnston et al. (28)	1997–1998	USA	Multiple EDs	Retrospective (review of medical records)	ED physician	Medical records
Jove et al. (29)	2008–2012	Spain	Single ED	Prospective (admissions to ED)	Neurologist	In person
Li et al. (30)	2010–2011	China	Single ED	Prospective (admissions to ED)	Neurologist	In person
Lim et al. (31)	2010–2012	Korea	Multiple EDs	Prospective (admissions to ED, Korean TIA Registry)	Neurologist	In person or phone
Nakajima et al. (32)	2002–2004	Japan	Specialist cardiovascular center	Prospective (admissions to cardiovascular center)	Neurologist	Medical records or Phone
Ohara et al. (33)	2008–2013	Japan	Hospital stroke center	Retrospective (review of stroke database records)	Stroke neurologist	In person
Ois et al. (34)	2004–2007	Spain	Single ED	Prospective (admissions to ED)	Neurologist	In person or Phone
Ong et al. (35)	2005–2006	Singapore	Single ED	Retrospective (ED database and medical records)	ED physician	Medical records
Perry et al. (36)	2006–2011	Canada	Multiple EDs	Prospective (admissions to ED)	ED, neurologists, residents	Phone
Purroy et al. (37)	2002–2005	Spain	Single ED	Prospective (admissions to ED)	Neurologist	In person
Purroy et al. (38)	2006–2009	Spain	Single ED	Prospective (admissions to ED)	Neurologist	In person
Purroy et al. (39)	2008–2009	Spain	Multiple stroke centers	Prospective (admissions to stroke center)	Stroke neurologist	In person
Tsivgoulis et al. (40)	2008–2009	Greece, Singapore	Multiple neurology departments	Prospective (admissions to ED)	Neurologist	In person
Zhao et al. (41)	2008–2011	China	Neurology department	Prospective (admissions to neurology)	Neurologist	Phone

*ED, Emergency Department*.

### Clinical Symptoms/Outcome Measures

We identified nine outcome measures related to motor impairment (a) motor lacunar symptom, (b) motor deficits, (c) motor weakness, (d) weakness, (e) unilateral weakness, (f) unilateral motor weakness, (g) focal weakness, (h) limb weakness, and (i) hemiparesis. The study authors defined these outcomes as motor weakness. We extracted data on all available motor outcome measures from each study. Only a few studies reported more than one outcomes (18, 27, 28, 32, 36, 38, 40). To prevent data biasing, we followed standard recommendations and selected one outcome measure per study that best represented motor weakness. Table 2 lists these outcome measures and other study characteristics.

**Table 2:** Characteristics of studies included in the meta-analysis.

Study name	Age	Sex	Sample size	Clinical symptom/outcome measure	Symptom to evaluation	Clinical evaluation	Neuroimaging evaluation	Neuroimaging findings	Recurrence time
Al-Khaled and Eggers (18)	70.6 ± 12.8	M = 1,122, F = 1,078	2,200	Unilateral Motor Weakness	2 days	ABCD^2^ score	MRI with DWI	–	During hospitalization
Ay et al. (19)	67.7 ± 14.7	M = 231, F = 246	477	Focal Weakness	1 day	ABCD^2^ score	MRI with DWI	DWI +ve = 136; DWI −ve = 318	7 days
Bray et al. (20)	73 ± 14.5	M = 49, F = 49	98	Unilateral Weakness	2 days	ABCD score	–	–	90 days
Chandratheva et al. (21)	72.5 ± 12.7	M = 219, F = 281	488	Unilateral Weakness	1 day	ABCD^2^ score	CT or MRI	–	1 day
Chen et al. (22)	–	–	199	Limb Weakness	–	–	CT or MRI	–	90 days
Coutts et al. (23)	Median 69 (27–99)	M = 293, F = 206	499	Motor Weakness	2 days	ABCD^2^ score	CT or MRI with DWI	CT +ve = 171; CT −ve = 328; DWI +ve = 243; DWI −ve = 256	7 days
Dai et al. (24)	62 ± 12.5	M = 486, F = 176	658	Motor Weakness	3 days	ABCD score	MRI with DWI	DWI +ve = 236; DWI −ve = 422	90 days
Fujinami et al. (25)	69 ± 13	M = 292, F = 172	464	Hemiparesis	7 days	ABCD^2^ score	MRI with DWI	DWI +ve = 96; DWI −ve = 368	During hospitalization
Gon et al. (26)	64 ± 15	M = 88 F = 51	139	Motor Weakness	7 days	ABCD^2^ score	MRI with DWI	DWI +ve = 53; DWI −ve = 86	14 days
Hayashi et al. (27)	66.6 ± 11.0	M = 44, F = 30	74	Hemiparesis	–	ABCD^2^ score	MRI with DWI	–	2 years
Johnston et al. (28)	Mean 72	M = 808, F = 899	1,707	Weakness	–	–	–	–	90 days
Jove et al. (29)	71.7 ± 10.8	–	293	Weakness	1 day	ABCD^2^ score	MRI with DWI		90 days
Li et al. (30)	67.5 ± 11.1	M = 70, F = 36	106	Motor Deficits	–	ABCD^2^ score	MRI with DWI		7 days
Lim et al. (31)	64.4 ± 11.8	M = 291, F = 209	500	Motor Weakness	2 days	ABCD^2^ score	MRI with DWI	DWI +ve = 140; DWI −ve = 335	90 days
Nakajima et al. (32)	65 ± 12	M = 81, F = 32	113	Hemiparesis	7 days	ABCD^2^ score	CT, MRI with DWI	DWI +ve = 39; DWI −ve = 74	90 days
Ohara et al. (33)	−	M = 263, F = 147	410	Motor Lacunar Symptom	2 days	ABCD^2^ score	MRI with DWI	DWI +ve = 119; DWI −ve = 291	7 days
Ois et al. (34)	−	−	221	Weakness	−	ABCD score	CT		90 days
Ong et al. (35)	61 ± 13.2	M = 293, F = 177	470	Unilateral Weakness	−	ABCD^2^ score	CT or MRI	−	90 days
Perry et al. (36)	68.0 ± 14.4	M = 1,930 F = 1,976	3,906	Weakness	<7 days	−	CT	CT +ve = 1,101	7 days
Purroy et al. (37)	70.8 ± 12	M = 230, F = 158	388	Weakness	1 day	ABCD^2^ score	CT	–	90 days
Purroy et al. (38)	69.3 ± 11.8	M = 156, F = 98	254	Motor Weakness	7 days	ABCD^2^ score	CT, MRI with DWI	DWI +ve = 117 DWI −ve = 137	90 days
Purroy et al. (39)	68.6 ± 13.1	M = 674, F = 463	1,137	Motor Weakness	2 days	ABCD^2^ score	CT, MRI with DWI	DWI +ve = 194 DWI −ve = 269	90 days
Tsivgoulis et al. (40)	60 ± 14	M = 82, F = 66	148	Unilateral Weakness	–	ABCD^2^ score	CT or MRI		90 days
Zhao et al. (41)	–	M = 119, F = 61	180	Weakness	–	–	–	–	90 days

*M, males; F, females; ABCD/ABCD^2^, Stroke Risk Score; MRI, magnetic resonance imaging; DWI, diffusion weighted imaging; CT, computerized tomography; Recurrence time is time from TIA symptom onset to stroke*.

### Data Synthesis and Analysis

**Figure 2:**
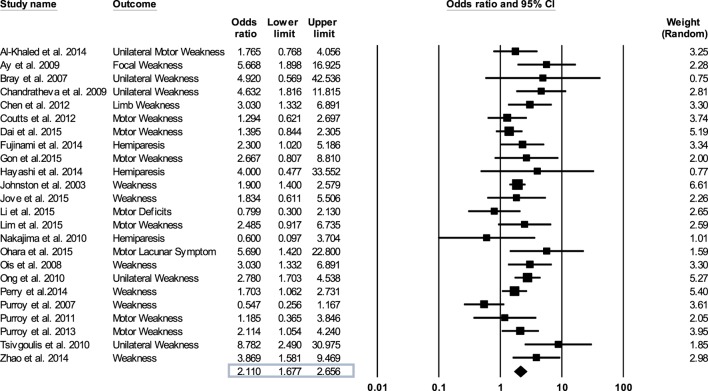
Forest plot derived from a random effects model. Each tick mark and line represents an individual odds ratio with a 95% confidence interval. The diamond shape at the bottom of the forest plot is the overall odds ratio (2.11) for all 24 studies.

We evaluated the quality of the studies included in the meta-analysis by the Downs and Black method (42). The Comprehensive Meta-Analysis Program was used to synthesize and analyze the data extracted from the TIA studies. This procedure involved entering the odds ratios, lower and upper limits, and confidence levels relating motor weakness to subsequent stroke from each study and then determining an overall odds ratio (Figure 2). For those studies that did not report an odds ratio (18–21, 23, 24, 26, 27, 29–31, 36–41), we computed the odd ratios as OR = (*A*/*B*)/(*C*/*D*). Here, *A* is the number of individuals with weakness and a recurrent stroke, *B* is the number of individuals with weakness without a recurrent stroke, *C* is the number of individuals without weakness with a recurrent stroke, and *D* is the number of individuals without weakness and without a recurrent stroke. We conducted sensitivity analysis to determine the extent to which the odds ratio was influenced by a particular study.

### Measuring Heterogeneity and Publication Bias

*I*^2^ was computed to determine the degree of variability across studies (43). We examined publication bias using (a) funnel plot asymmetry, (b) Duval and Tweedie’s trim and fill procedure that creates a funnel plot with imputed values inserted as close approximations to a completely unbiased distribution, and (c) fail-safe *N* analysis that uses the probability value of the cumulative odds ratio to determine the number of studies required to render the odds ratio insignificant.

## Results

### Characteristics of the Included Studies

Twenty-four studies with 15,129 participants qualified for inclusion in the meta-analysis. Figure 1 shows the step-by-step procedure of identifying studies that satisfied the criteria for inclusion in this meta-analysis. The studies were conducted between 1997 and 2013, in North America (*N* = 4, participants = 6,589), Australia (*N* = 1, participants = 98), Asia (*N* = 11, participants = 3,313), and Europe (*N* = 8, participants = 5,129). One study was conducted in two locations (40). TIA was diagnosed by a neurologist in the majority of the studies (*N* = 19). The duration between symptom onset to clinical evaluation ranged from 1 to 7 days. The clinical evaluation of motor impairment included ABCD or ABCD^2^ score (*N* = 20). The neuroimaging evaluations included magnetic resonance imaging (MRI; *N* = 18). The recurrence time from the onset of TIA symptom to occurrence of stroke varied from 1 day up to 2 years. Table 3 reports the study quality. All studies had a minimum quality score of 11 out of 17.

**Table 3:** Quality scores using Downs and Black scale: checklist for measuring study quality.

Study name	1	2	3	5	6	7	9	10	11	12	13	16	18	20	25	26	Quality score
Al-Khaled and Eggers (18)	1	1	1	0	1	1	0	1	1	0	1	1	1	1	0	0	11
Ay et al. (19)	1	1	1	0	1	1	1	1	1	1	1	1	1	1	0	1	14
Bray et al. (20)	1	1	1	1	1	1	1	0	1	1	1	1	1	1	1	1	15
Chandratheva et al. (21)	1	1	1	2	1	1	0	1	1	0	1	1	1	1	1	0	14
Chen et al. (22)	1	1	1	1	1	1	1	0	1	1	1	1	1	1	1	1	15
Coutts et al. (23)	1	1	1	2	1	1	1	1	0	0	1	1	1	1	1	1	15
Dai et al. (24)	1	1	1	1	1	1	1	1	1	1	1	1	1	1	0	1	15
Fujinami et al. (25)	1	1	1	2	1	1	1	1	1	1	1	1	1	1	0	1	16
Gon et al. (26)	1	1	1	1	1	1	0	0	1	0	1	1	1	1	0	0	11
Hayashi et al. (27)	1	1	1	1	1	1	0	1	0	0	1	1	1	1	0	0	11
Johnston et al. (28)	1	1	1	0	1	1	1	1	1	1	1	1	1	1	0	1	14
Jove et al. (29)	1	1	1	2	1	1	0	1	1	0	1	1	1	1	1	0	14
Li et al. (30)	1	1	1	0	1	1	1	1	1	1	1	1	1	1	0	1	14
Lim et al. (31)	1	1	1	2	1	1	1	1	1	1	1	1	1	1	0	1	16
Nakajima et al. (32)	1	1	1	1	1	1	1	1	1	1	1	1	1	1	1	1	16
Ohara et al. (33)	1	1	1	1	1	1	1	1	1	1	1	1	1	1	1	1	16
Ois et al. (34)	1	1	1	2	1	1	0	1	1	0	1	1	1	1	1	0	14
Ong et al. (35)	1	1	1	2	1	1	0	0	1	0	1	1	1	1	0	0	12
Perry et al. (36)	1	1	1	2	1	1	0	1	1	0	1	1	1	1	1	1	15
Purroy et al. (37)	1	1	1	1	1	1	1	1	0	0	1	1	1	1	0	1	13
Purroy et al. (38)	1	1	1	1	1	1	1	1	0	0	1	1	1	1	0	1	13
Purroy et al. (39)	1	1	1	2	1	1	1	1	1	1	1	1	1	1	0	1	16
Tsivgoulis et al. (40)	1	1	1	2	1	1	1	1	1	1	1	1	1	1	1	1	17
Zhao et al. (41)	1	1	1	2	1	1	1	1	1	1	1	1	1	1	1	0	16

*Questions 4, 8, 14, 15, 17, 19, 21, 22, 23, 24, and 27 were not used because the studies included in the meta-analysis were not interventional. Questions 1, 2, 3, 5, 6, 7, 9, 10, 11, 12, 13, 16, 18, 20, 25, and 26 add to a maximum total possible score of 17*.

### Motor Impairments in TIA and Subsequent Stroke

Figure 2 shows forest plot of the odds ratio across individual studies. The odds ratio was computed using random effects model to determine the relation between the presence of motor impairments during a TIA and the chances of a subsequent stroke. The model revealed a pooled odds ratio of 2.11 (95% CI, 1.67–2.65; *p* = 0.000). Figure 2 shows the overall odds ratio to the right of the vertical line of no effect (1.00), indicating that the odds of a subsequent stroke are doubled in individuals who experience motor impairment during a TIA. The sensitivity analysis revealed that the odds of a subsequent stroke did not alter considerably with the exclusion of individual studies. Further, when studies with highest risk of bias (quality scores < 14) (18, 26, 27, 35, 37, 38) were excluded the overall odds ratio improved to 2.12 (95% CI, 1.58–2.84; *p* = 0.000).

### Heterogeneity

**Figure 3:**
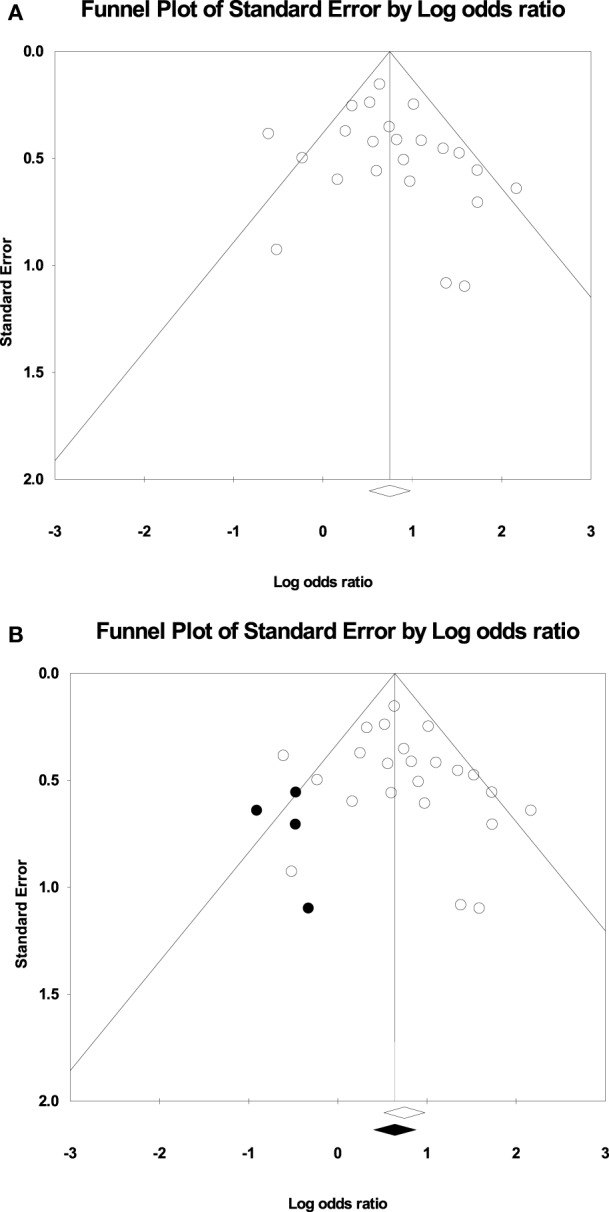
**(A)** Funnel plot of the studies for the random effects model: The *x*-axis is the log odds ratio, and the *y*-axis is the standard error associated with each study. **(B)** Funnel plot with imputed studies. Open circles represent the 24 original studies whereas black circles represent imputed studies.

Measurements of heterogeneity revealed an *I^2^* of 45.85 (*p* = 0.008). Because of this relatively large proportion of dispersion in the TIA studies, we conducted a random effects meta-analysis. Plotting the log odds ratio as a function of standard error revealed a symmetrical funnel plot (Figure 3A). This symmetrical funnel plot represents an unbiased summary effect. The majority of studies congregating on the top half of the funnel indicate the large sample sizes of the studies and a more precise estimate of the odds ratio with a smaller standard error.

### Publication Bias

Figure 3B shows the funnel plot with imputed studies using Duval and Tweedie’s trim and fill technique (43). Only four studies were imputed on the left side of the plot to achieve symmetry, signifying an unbiased effect. The black diamond on the *x*-axis indicating the recalculated log odds ratio is highly similar to the original log odds ratio. The fail-safe analysis revealed that 462 null effect findings were necessary to lower the cumulative odds ratio of 2.11 to an insignificant level. Therefore, the odds ratio associating the presence of motor impairments in TIA with a recurrent stroke offers a robust finding.

## Discussion

The purpose of the current meta-analysis was to examine whether the odds of a recurrent stroke are greater in TIA individuals who experience motor impairments compared with those who do not experience motor impairments. Our meta-analysis included 24 high-quality studies that examined motor impairments in TIA and a subsequent stroke in 15,129 individuals. The novel finding from this meta-analysis is that TIA individuals with motor impairments are twice as likely to experience a stroke as compared with those who have no motor impairments. Thus, motor impairments during TIA are a significantly high-risk clinical characteristic for a recurrent stroke.

Traditionally, stroke risk is determined using ABCD^2^ score in the clinical settings (40). ABCD^2^ score is a cumulative score derived from multiple TIA characteristics including motor and speech impairment (44). However, the evidence supporting ABCD^2^ score for predicting stroke risk remains inconclusive. While previous studies showed that TIA individuals with ABCD^2^ score of >3 were at a high early risk of stroke (44, 45), more recent studies have questioned the reliability of the score in distinguishing the low and high risk of stroke recurrence (46, 47). The current meta-analysis suggests that the odds of a recurrent stroke are doubled in individuals who experience motor impairments during a TIA. These findings complement the clinical ABCD^2^ score that ascribes twice the predictive weight to unilateral motor weakness than speech impairment. Further, our findings are consistent with a recent study in urgent care setting that reported greater risk of stroke in TIA individuals with unilateral motor weakness (48). Regardless of the predictive capacity of the cumulative score, emerging evidence clearly suggests that the presence of motor impairments during a TIA in itself is a compelling determinant of the increased odds of a subsequent stroke.

One question concerns why are the odds of a subsequent stroke increased when motor impairments are present during a TIA. Clinical reports indicate that motor impairments during a TIA are often concomitant with structural brain abnormalities (49). Additionally, the presence of acute brain lesions detected as positive diffusion-weighted imaging (DWI) significantly increases the probability of a subsequent stroke following TIA (50, 51). Thus, one possibility is that perhaps motor impairments during a TIA increase the odds of a subsequent stroke because of an underlying ischemic lesion. Future studies are needed to clearly identify the mechanisms underlying increased stroke risk in TIA individuals with motor impairments.

Current standard of diagnostic protocol for the evaluation of motor deficits in TIA focuses primarily on the assessment of motor weakness. However, individuals with TIA experience multiple motor impairments including reduced coordination, impaired motor control, gait disturbance, dysarthria, and ataxia (36, 52). Thus, the clinical diagnosis of motor weakness may be inadequate for identifying residual motor deficits following TIA (53, 54). Clearly then, the absence of comprehensive motor examination for TIA individuals potentially understates the extent to which motor impairments are prevalent following TIA.

Further, initial symptoms are considered to be resolved shortly after the onset of TIA. Contrary to this conventional view point, recent evidence suggests that individuals experience subtle problems in functional activities of daily living up to 6 months after TIA (55, 56). This is corroborated by a recent report indicating that TIA individuals are more likely to consult clinicians for residual impairments than age-matched controls (54). Furthermore, about 50% of the individuals who have a TIA require rehabilitation to resolve the residual impairments that affect everyday function (55, 57). Thus, the supposed transient nature of initial motor impairments may not be so transient, pointing to the pressing need for a systematic evaluation and restoration of motor abilities beyond the clinically determined weakness in individuals with TIA.

### Considerations and Future Directions

Few limitations of the meta-analysis findings require consideration. First, our findings may be modest in determining on the true impact of motor impairments on the odds of a recurrent stroke. This is because we extracted the data on most widely reported motor impairment, i.e., motor weakness, despite multiple motor symptoms associated with TIA. In addition, the relatively large variability in the time from symptom onset to evaluation at hospital admission contributes to the unrecognized diagnosis of motor impairments due to symptom recession or missed reporting by individuals who sought delayed medical attention. Further, the tissue-based definition for TIA diagnosis recommends the use of neuroimaging for distinguishing a minor stroke from a TIA. However, the majority of studies included in this meta-analysis based their diagnosis of TIA on the time-based definition. Finally, these findings do not understate the importance of careful clinical follow-up in TIA individuals without motor impairments. Rather, they point to the need for differential clinical care and rehabilitation of TIA individuals who experience motor impairments. In summary, the findings from the current meta-analysis offer strong evidence favoring significantly greater risk of a subsequent stroke in TIA individuals who experience motor impairments.

Given the findings from this meta-analysis, a comprehensive clinical evaluation of motor impairments is highly recommended. For example, examining motor output variability, accuracy, symmetry, and coordination deficits will provide more meaningful insights into the overall decline in motor ability following TIA. Our past work shows that motor control abilities are more predictive of everyday function in older adults than motor weakness (58). Thus, a comprehensive motor examination following TIA will further our understanding of the impact of motor impairments on functional tasks of daily living and improve the treatment and rehabilitation of residual motor impairments.

## Conclusion

Transient ischemic attack individuals with motor impairments are at significantly greater risk for a recurrent stroke. The current evidence for motor impairments following TIA relies exclusively on the clinical reports of motor weakness. A comprehensive examination of motor impairments in TIA will enhance TIA prognosis and restoration of residual motor impairments.

## Author Contributions

NL, JH, and EC were involved in conceptualization, design, data coding, extraction. NL, JH, SE, EC participated in the interpretation of the meta-analytic results. NL and JH wrote the first draft of the manuscript. NL, JH, SE, and EC revised and approved the final version of the manuscript.

## Conflict of Interest Statement

The authors declare that the research was conducted in the absence of any commercial or financial relationships that could be construed as a potential conflict of interest.
